# APOBEC3B gene expression as a novel predictive factor for pathological complete response to neoadjuvant chemotherapy in breast cancer

**DOI:** 10.18632/oncotarget.25495

**Published:** 2018-07-17

**Authors:** Yoshitaka Fujiki, Yutaka Yamamoto, Aiko Sueta, Mutsuko Yamamoto-Ibusuki, Lisa Goto-Yamaguchi, Mai Tomiguchi, Takashi Takeshita, Hirotaka Iwase

**Affiliations:** ^1^ Department of Breast and Endocrine Surgery, Kumamoto University Graduate School of Medical Sciences, Chuo-Ku, Kumamoto 860-8556, Japan; ^2^ Department of Molecular-Targeting Therapy for Breast Cancer, Kumamoto University Hospital, Chuo-Ku, Kumamoto 860-8556, Japan

**Keywords:** APOBEC3B, breast cancer, neoadjuvant chemotherapy, predictive factor, pathological complete response

## Abstract

**Background:**

Apolipoprotein B mRNA editing enzyme catalytic polypeptide-like 3B (APOBEC3B) is a gene editing enzyme with cytidine deaminase activity and high expression of its mRNA in breast tumors have been shown to be associated with progressive cases and poor prognosis. In this study, we aimed to examine the relationship between the expression of APOBEC3B and the effect of neoadjuvant chemotherapy (NAC) using pretreatment biopsy tissue, and examined whether the expression of APOBEC3B influenced chemotherapy efficacy.

**Methods:**

We retrospectively selected a total of 274 patients with primary breast cancer who received NAC in more than 4 courses and underwent surgery at our institute. We assessed the expression of APOBEC3B mRNA using pretreatment biopsy specimens of NAC by quantitative real-time PCR (qRT-PCR) and examined the relationship between APOBEC3B mRNA expression and sensitivity to chemotherapy using pathological complete response (pCR) as an indicator. Further, we assessed the prognostic value of APOBEC3B in the patients receiving NAC.

**Results:**

APOBEC3B mRNA expression levels were successfully assessed in 173 (63.1%) of the 274 specimens. The total pCR rate was 36.4% (n = 63). An association between APOBEC3B expression levels and pCR was observed (Wilcoxon test, P ≤ 0.0001). The patients were divided into two groups, low (n = 66) and high (n = 107), according to the APOBEC3B expression levels, using the cut-off value calculated by the receiver operating characteristics (ROC) curve for pCR. The rate of pCR was significantly higher among the patients in the high group than among those in the low group (47.7% vs 18.2%, P ≤ 0.0001). High APOBEC3B expression was significantly associated with high nuclear grade (P = 0.0078), high Ki-67 labeling index (P = 0.0087), estrogen receptor (ER) negativity (P ≤ 0.0001) and human epidermal growth factor receptor 2 (HER2) negativity (P = 0.032). Tumor size (P = 0.011), ER (P ≤ 0.0001), HER2 (P = 0.0013) and APOBEC3B expression (P = 0.037) were independent predictive factors for pCR in multivariate analysis. However, there was no association between APOBEC3B expression and prognosis.

**Conclusions:**

Our study showed that APOBEC3B mRNA expression correlated with sensitivity to NAC in breast cancer patients. In contrast to previous studies, APOBEC3B mRNA expression was not associated with breast cancer prognosis in patients receiving NAC.

## INTRODUCTION

Recent studies have shown that many cancers are caused by somatic mutations, which occur randomly in the DNA over the course of an individual’s lifetime [1, 2]. From several hundreds to thousands of mutations, with the prevalence of somatic mutations, have been reported in various cancers [2–8]. To date, a number of genome sequencing studies have revealed that many cancers, including breast cancer, have somatic mutation spectra, mainly including base rearrangement from cytosine (C) to thymine (T) (complementary chain, guanine [G] →adenine [A]) [4, 5, 9, 10]. Most of these mutations are sometimes clustered [6, 11].

APOBEC3B is shown to be significantly contributed to a source of the above somatic mutation for several types of cancer including breast cancer [9, 10, 12–15]. APOBEC3B mutation signature is specifically enriched (C to T transition) in six types of cancers, including cervix, bladder, lung (adeno and squamous cell), head and neck, and breast cancers [9, 10]. APOBEC3B is a gene editing enzyme having cytidine deaminase activity, and the protein family comprises eleven members in humans: activation-induced cytidine deaminase (AID) and APOBEC1 (genes located on chromosome 12), APOBEC2 (gene located on chromosome 6), seven APOBEC3 proteins (APOBEC3A/B/C/D/F/G/H; genes located on chromosome 22) and APOBEC4 (gene located on chromosome 1) [16, 17]. APOBEC family members have been identified as intracellular antiviral factors, are normally part of the innate immune system and protect against viral pathogens (retrovirus and retrotransposon propagation, such as restricting HIV-1 viral reverse transcription) [17, 18]. However, specific mutations in cancer (APOBEC mutagenesis) may mainly induce C to T mutation pattern and have a role in carcinogenesis [12, 19, 20]. This mutagenesis is due to cytidine deaminase activity of APOBEC3B, which deaminates cytosine in DNA and RNA and leads to C to T transition mutation.

In breast cancer, the C to T transition mutation of TCA or TCT sequences by APOBEC3B has been observed frequently [6, 9, 10, 19]. Several studies showed that the expression levels of APOBEC3B in tumor tissue were higher compared with normal tissue [19, 21, 22]. In addition, APOBEC3B may contribute to canceration and progression of breast cancer due to accumulation of mutations. High expression of APOBEC3B has been reported in advanced cases and cases with poor prognosis [10, 23]. Recently, it has also been shown that APOBEC3B influences metastasization, prognosis and endocrine therapy resistance in estrogen receptor (ER) -positive breast cancer [22–25].

Despite these findings, there are no reports comparing APOBEC3B and therapeutic effect of chemotherapy. Previous studies have shown that NAC for primary breast cancer has the same recurrence suppression effect as postoperative chemotherapy [26, 27]. Therefore, NAC has been one of the standard treatment strategies for breast cancer patients. In particular, NAC has several advantages, such as tumor shrinkage, improvement of surgical outcome and monitoring of response to systemic therapy. The patients with acquired pCR by NAC have a good prognosis, and pCR is considered as a surrogate prognostic marker for breast cancer [28–34]. It is very important to examine the relationship between pCR and APOBEC3B in NAC. In the present study, we examined the relationship between APOBEC3B mRNA expression and sensitivity to NAC or prognosis of patients receiving NAC by performing real-time quantitative reverse transcription PCR (RT-qPCR) on formalin-fixed paraffin-embedded (FFPE) specimens.

## RESULTS

### Patient characteristics and APOBEC3B gene expression in FFPE breast cancer specimens

One hundred and seventy-three FFPE specimens (63.1%) of the total 274 cases were shown individually to contain detectable levels of all 4 housekeeping genes and APOBEC3B gene at a Ct < 40 and were selected for this study. The remaining 101 samples were considered technical failures because RNA extraction was not successful in 36 samples, 13 samples had 1 or 2 abnormal Ct values for housekeeping genes, and 52 samples had abnormal Ct values for APOBEC3B gene (Supplementary Figure 1). The median relative quantification of APOBEC3B mRNA expression in the FFPE samples was 0.016 (range 0.00023–0.61). The clinicopathological factors of the 173 cases are summarized in the Supplementary Table 1. The median age at diagnosis was 53 years (range 24–78). pCR rate of 173 cases was 36.4% (n = 63). The patients were followed up postoperatively every 3 months if they had no recurrence. The median follow-up period was 57 months (range 4–158). Tumor subtypes were defined according to the expression of ER, progesterone receptor (PgR), and human epidermal growth factor receptor 2 (HER2); luminal (ER+ and/or PgR+, HER2-), luminal-HER2 (ER+ and/or PgR+, HER2+), HER2-enriched (ER- and PgR-, HER2+), and triple-negative (ER-, PgR- and HER2-).

### Association of APOBEC3B mRNA expression with clinicopathological characteristics

We examined the relationship between APOBEC3B mRNA expression and clinicopathological features (Table 1). APOBEC3B mRNA expression positively correlated with pCR (P ≤ 0.0001). Moreover, we examined the association A3B mRNA expression with the four main molecular subtypes (luminal, luminal-HER2, HER2-enriched and triple negative) (Supplementary Figure 2). High A3B mRNA expression was related to triple negative subtype (P ≤ 0.0001). We divided the samples into two groups using the cut-off value of the ROC analysis of pCR; 107 cases (61.8%) were defined as the high expression group and 66 cases (38.2%) were defined as the low expression group. The high expression group had higher rate of pCR (47.7%: 51/107) than the low expression group (18.2%: 12/66) (P ≤ 0.0001). Higher levels of APOBEC3B mRNA were associated with high nuclear grade (grade 3; P = 0.0027), high Ki67 labeling index (≥20%; P = 0.0022), negative ER status (P ≤ 0.0001) and subtype (triple-negative; P ≤ 0.0001). Likewise, the high expression group of APOBEC3B classification also positively correlated with the corresponding parameters. There were no correlations between APOBEC3B expression level and age, menopausal status, or nodal status.

**Table 1:** Relationship between APOBEC3B mRNA expression and clinicopathological characteristics

Characteristics	Number of patients (%)	APOBEC3B mRNA expression levels				
		Median (25%, 75%)	P-value	High (n = 107)	Low (n = 66)	P-value
Age						
<50	71 (41.0%)	0.018 (0.0071, 0.037)	0.25	48 (44.9%)	23 (34.9%)	0.19
≥50	102 (59.0%)	0.014 (0.0054, 0.048)		59 (55.1%)	43 (65.1%)	
Menopause						
Premenopausal	72 (41.6%)	0.017 (0.0061, 0.31)	0.95	48 (44.9%)	24 (36.4%)	0.27
Postmenopausal	101 (58.4%)	0.016 (0.0058, 0.047)		59 (55.1%)	42 (63.6%)	
Tumor size (mm)						
<20	29 (16.8%)	0.013 (0.0044, 0.028)	0.37	17 (15.9%)	12 (18.2%)	0.69
≥20	144 (83.2%)	0.017 (0.0062, 0.040)		90 (84.1%)	54 (81.8%)	
Nuclear Grade						
1	34 (19.7%)	0.0092 (0.0054, 0.019)	0.0027	15 (14.0%)	19 (28.8%)	0.0078
2	63 (36.4%)	0.013 (0.0034, 0.033)		36 (33.6%)	27 (40.9%)	
3	76 (43.9%)	0.023 (0.0091, 0.070)		56 (52.3%)	20 (30.3%)	
Ki67 labeling index						
<20	26 (15.0%)	0.0081 (0.0026, 0.017)	0.0022	10(9.4%)	16 (24.2%)	0.0087
≥20	147 (85.0%)	0.018 (0.0070, 0.045)		97 (90.7%)	50 (75.8%)	
Nodal status						
Negative	51 (29.5%)	0.017 (0.0058, 0.039)	0.88	33 (30.8%)	18 (27.3%)	0.62
Positive	122 (70.5%)	0.016 (0.0058, 0.040)		74 (69.2%)	48 (72.7%)	
Stage						
I	12 (7.8%)	0.0083 (0.0024, 0.090)	0.39	5 (4.7%)	7 (10.6%)	0.0042
II	108 (64.1%)	0.017 (0.0075, 0.039)		77 (72.0%)	31 (47.0%)	
III	53 (28.1%)	0.0095 (0.0095, 0.035)		25 (13.4%)	28 (42.4%)	
ER						
-	70 (40.5)	0.032 (0.011, 0.073)	<0.0001	56 (52.3%)	14 (21.2%)	<0.0001
+	103 (59.5%)	0.010 (0.0041, 0.22)		51 (47.7%)	52 (78.8%)	
PgR						
-	85 (49.1%)	0.029 (0.010, 0.072)	<0.0001	65 (60.8%)	20 (30.3%)	<0.0001
+	88 (50.9%)	0.0095 (0.0041, 0.021)		42 (39.3%)	46 (69.7%)	
HER2						
-	120 (69.3%)	0.014 (0.0047, 0.038)	0.082	68 (63.6%)	52 (78.8%)	0.032
+	53 (30.6%)	0.020 (0.0088, 0.042)		39 (36.5%)	14 (21.2%)	
Tumor subtype						
Luminal	79 (45.6%)	0.0090 (0.0035, 0.021)	<0.0001	35 (32.7%)	44 (66.7%)	0.0001
Luminal-HER2	24 (13.9%)	0.018 (0.0075, 0.061)		16 (15.0%)	8 (12.1%)	
HER2-enriched	29 (16.8%)	0.020 (0.010, 0.042)		23 (21.5%)	6 (9.1%)	
Triple negative	41 (23.7%)	0.044 (0.014, 0.12)		33 (30.8%)	8 (12.1%)	
pCR status						
pCR	63 (36.4%)	0.021 (0.0028, 0.61)	<0.0001	51 (47.7%)	12 (18.2%)	<0.0001
non-pCR	110 (63.6%)	0.011 (0.00023, 0.34)		56 (52.3%)	54 (81.8%)	

Abbreviations: ER; estrogen receptor, PgR; progesteron receptor, HER2; human epidermal growth factor 2, pCR; pathological complete response. Luminal (ER+ and/or PgR+, HER2-), Luminal-HER2 (ER+ and/or PgR+, HER2+), HER2-enriched (ER- and PgR-, HER2+), Triple negative (ER-, PgR- and HER2-).

### Univariate and multivariate analyses for predictive pCR

**Figure 1:**
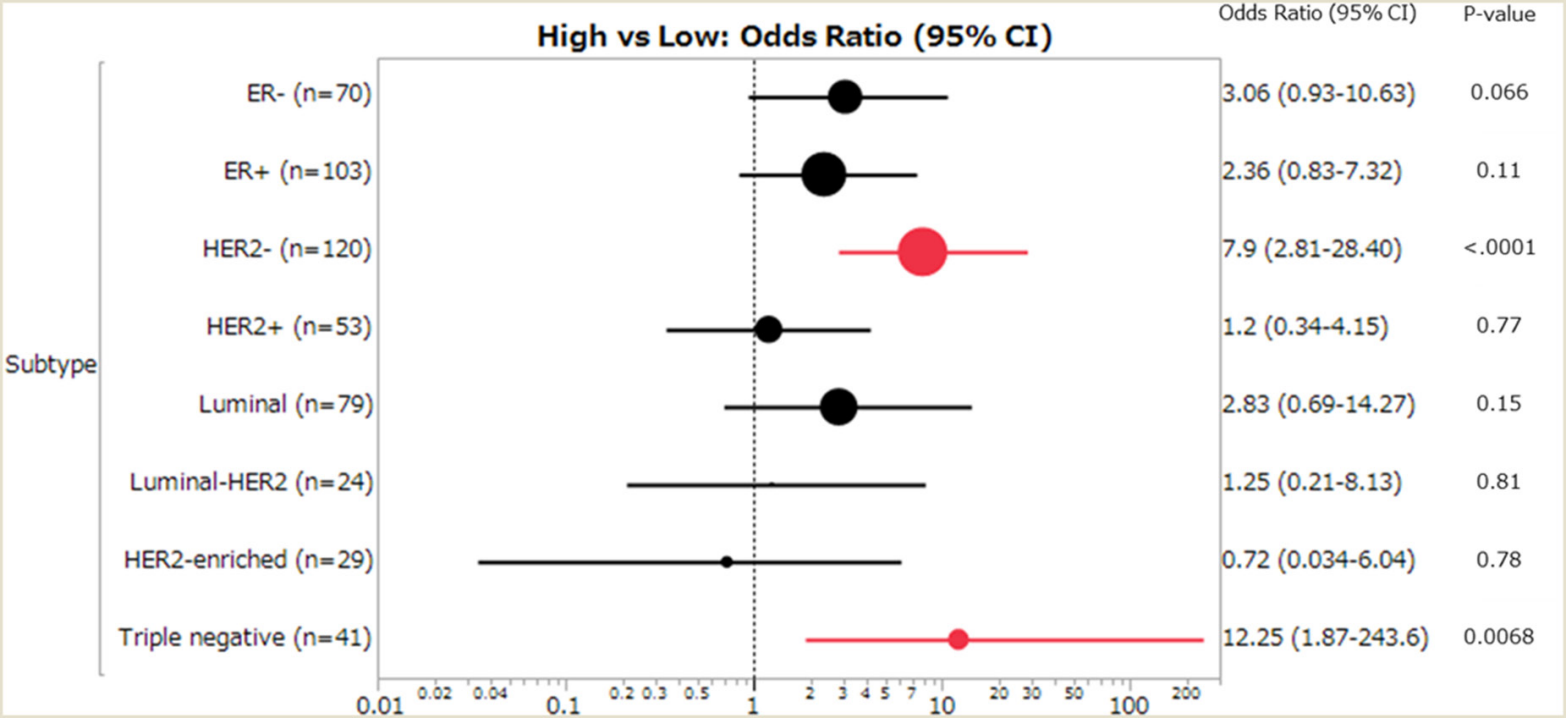
Forest plot of the odds ratios (ORs) and 95% confidence intervals (CIs) on the association between pCR and APOBEC3B mRNA expression (high vs low) The size of the black circle is proportional to the sample size. The horizontal line shows 95% CI of the OR. Luminal (ER+ and/or PgR+, HER2-), Luminal-HER2 (ER+ and/or PgR+, HER2+), HER2-enriched (ER- and PgR-, HER2+), Triple negative (ER-, PgR- and HER2-).

We examined the relationship between pCR status and clinicopathological characteristics (Table 2). The patients with small tumor size (P = 0.024), high nuclear grade (P = 0.0038), negative ER and PgR status (P ≤ 0.0001), positive HER2 status (P = 0.032) and subtype (HER2-enriched, triple-negative; P ≤ 0.0001) were more likely to achieve pCR. Next, we evaluated the association between APOBEC3B and pCR when stratified by each breast cancer subtype (Figure 1). We observed a significant correlation between APOBEC3B classification and pCR in two groups (negative HER2; OR = 7.9, P ≤ 0.0001, triple-negative; OR = 12.25, P = 0.0068).

**Table 2:** Relationship between pCR status and clinicopathological characteristics

Characteristics	Number of patients (%)	pCR status		
		non-pCR (n = 110)	pCR (n = 63)	P-value
Age				
<50	71 (41.0%)	47 (42.7%)	24 (38.1%)	0.55
≥50	102 (59.0%)	63 (57.3%)	39 (61.9%)	
Menopause				
Premenopausal	72 (41.6%)	51 (46.3%)	21 (33.3%)	0.092
Postmenopausal	101 (58.4%)	59 (53.6%)	42 (66.7%)	
Tumor size (mm)				
<20	29 (16.8%)	13 (11.8%)	16 (25.4%)	0.024
≥20	144 (83.2%)	97 (88.2%)	47 (74.6%)	
Nuclear Grade				
1	34 (19.7%)	6 (9.5%)	28 (25.5%)	0.0038
2	63 (36.4%)	20 (31.8%)	43 (39.1%)	
3	76 (43.9%)	37 (58.7%)	39 (35.4%)	
Ki67 labeling index				
<20	26 (15.0%)	24 (21.8%)	2 (3.2%)	0.0003
≥20	147 (85.0%)	86 (78.3%)	61 (96.8%)	
Nodal status				
Negative	51 (29.5%)	24 (21.8%)	27 (42.9%)	0.0038
Positive	122 (70.5%)	86 (78.2%)	36 (57.1%)	
Stage				
I	12 (6.9%)	5 (4.6%)	7 (11.1%)	0.0026
II	108 (62.4%)	62 (56.4%)	46 (73.0%)	
III	53 (30.6%)	43 (39.0%)	10 (15.9%)	
ER				
-	70 (40.5)	25 (22.7%)	45 (71.4%)	<0.0001
+	103 (59.5%)	85 (77.3%)	18 (28.6%)	
PgR				
-	85 (49.1%)	35 (31.8%)	50 (79.4%)	<0.0001
+	88 (50.9%)	75 (68.2%)	13 (20.6%)	
HER2				
-	120 (69.3%)	89 (80.9%)	31 (49.2%)	0.032
+	53 (30.6%)	21 (19.1%)	32 (50.8%)	
Tumor subtype				
Luminal	79 (45.6%)	70 (63.6%)	9 (14.3%)	<0.0001
Luminal-HER2	24 (13.9%)	15 (13.6%)	9 (14.3%)	
HER2-enriched	29 (16.8%)	6 (5.5%)	23 (36.5%)	
Triple negative	41 (23.7%)	19 (17.3%)	22 (34.9%)	

Abbreviations: ER; estrogen receptor, PgR; progesteron receptor, HER2; human epidermal growth factor 2, pCR; pathological complete response. Luminal (ER+ and/or PgR+, HER2-), Luminal-HER2 (ER+ and/or PgR+, HER2+), HER2-enriched (ER- and PgR-, HER2+), Triple negative (ER-, PgR- and HER2-).

We evaluated the contribution of clinical variables at baseline to pCR prediction using logistic regression analysis (Table 3). Small tumor size, no involvement of axillary lymph nodes, high nuclear grade, low stage, negative ER and PgR status, positive HER2 status, high Ki67 labeling index (≥20%) and high APOBEC3B status were all significant in univariate analysis. In multivariate analysis, ER status (P ≤ 0.0001), HER2 status (P = 0.0013), and APOBEC3B classification (P = 0.037) remained significant and were independent predictive factor for pCR. Next, we evaluated the correlation between APOBEC3B mRNA expression and pCR in HER2- and triple-negative subtypes. APOBEC3B classification remained significant and was an independent predictive factor for pCR in both subtypes (Table 3).

**Table 3:** Univariate and multivariate analysis of factors associated with pCR

Factors		Univariate analysis			multivariate analysis		
		OR	95% CI	P-value	OR	95% CI	P-value
All cases (n = 173)							
Age	<50 vs ≥50	0.82	0.44–1.55	0.55			
Menopause	Pre vs Post	0.58	0.30–1.10	0.092			
Tumor size (mm)	<20 vs ≥20	2.54	1.13–5.80	0.024	3.86	1.36–11.9	0.011
Nodal status	Negative vs Positive	2.68	1.37–5.31	0.0038	1.67	0.55–3.51	0.48
Nuclear Grade	1,2 vs 3	0.39	0.20–0.72	0.003	1.15	0.48–2.83	0.76
Stage	I, II vs III	0.29	0.13–0.62	0.001	2.39	0.87–7.11	0.094
ER status	- vs +	8.5	4.27–17.6	<0.0001	5.74	2.46–14.1	<0.0001
PgR status	- vs +	8.2	4.07–17.7	<0.0001			
HER2 status	- vs +	0.23	0.11–0.45	<0.0001	0.28	0.11–0.59	0.0013
Ki67 labeling index	<20 vs ≥20	0.11	0.027–0.52	0.0003	0.34	0.036–1.23	0.094
APOBEC3B mRNA	High vs Low	4.1	1.97–8.52	<0.0001	2.7	1.1–7.0	0.037
HER2- cases (n = 120)							
Age	<50 vs ≥50	1.15	0.50–2.64	0.73			
Menopause	Pre vs post	0.85	0.36–1.92	0.69			
Tumor size (mm)	<20 vs ≥20	3.9	1.48–10.4	0.0064	5.65	1.59–23.2	0.007
Nodal status	Negative vs Positive	3.03	1.27–7.30	0.013	1.24	0.34–4.34	0.73
Nuclear Grade	1, 2 vs 3	0.25	0.10–0.59	0.0013	1.26	0.35–5.00	0.73
Stage	I, II vs III	5.03	1.78–18.1	0.0015	4.02	0.99–20.4	0.0509
ER status	- vs +	9	3.68–23.8	<0.0001	6.43	1.92–24.4	0.0022
PgR status	- vs +	6.97	2.86–18.5	<0.0001			
Ki67 labeling index	<20 vs ≥20	0.21	0.032–0.78	0.017	0.39	0.045–2.32	0.31
APOBEC3B mRNA	High vs Low	7.9	2.81–28.4	<0.0001	7.24	1.83–39.1	0.0037
Triple negative cases (n = 41)							
Age	<50 vs ≥50	1.6	0.43–6.46	0.49			
Menopause	Pre vs post	1.6	0.43–6.46	0.49			
Tumor size(mm)	<20 vs ≥20	1.41	0.33–6.44	0.024	4.63	0.68–92.8	0.13
Nodal status	Negative vs Positive	1.38	0.40–4.84	0.61			
Nuclear Grade	1,2 vs 3	1.05	0.26–4.37	0.95			
Stage	I, II vs III	2.63	0.65–11.9	0.18			
Ki67 labeling index	<20 vs ≥20	0.86	0.032–22.7	0.92			
APOBEC3B mRNA	High vs Low	12.25	1.87–243.6	0.0068	26.29	2.80–915.6	0.0021

Abbreviations: ER; estrogen receptor, PgR; progesteron receptor, HER2; human epidermal growth factor 2, pCR; pathological complete response, OR; odds ratio, CI; confidence interval, Pre; premenopausal, Post; postmenopausal. Triple negative; ER-, PgR- and HER2-.

### Prognostic relevance of APOBEC3B mRNA expression and pCR

**Figure 2:**
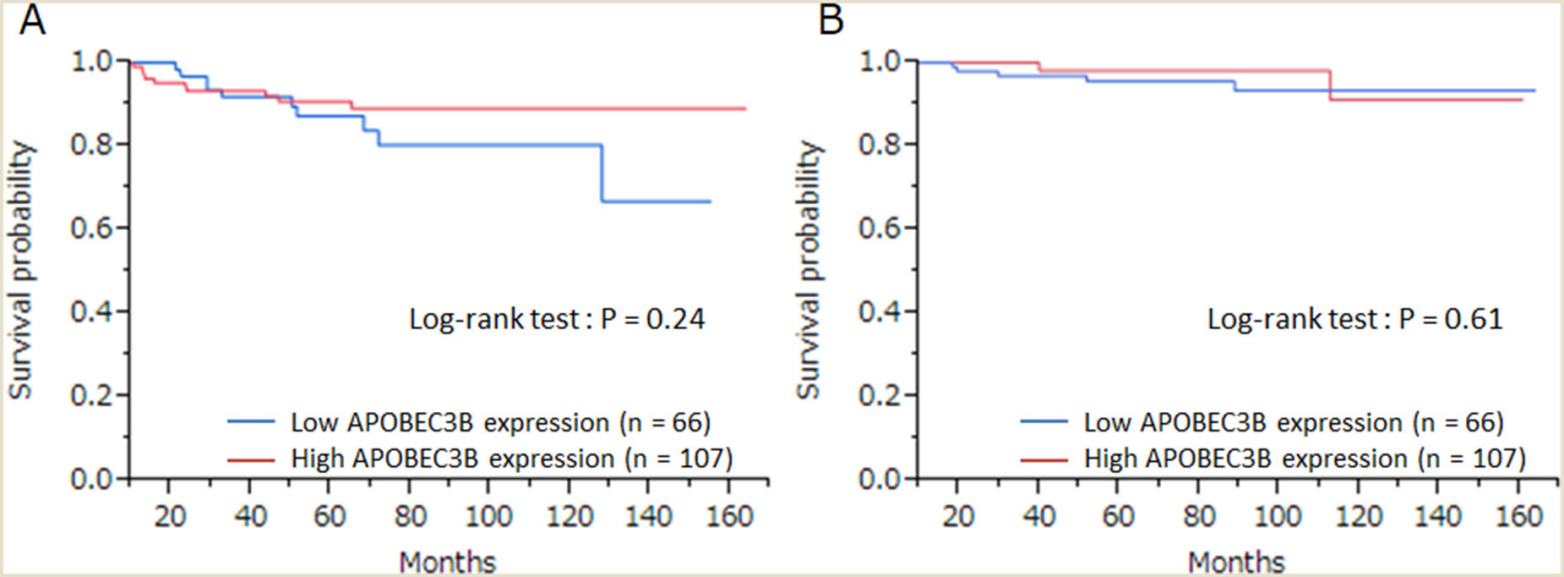
The relationship between APOBEC3B mRNA expression and prognosis Kaplan–Meier plots showing the association of APOBEC3B mRNA expression with **(A)** relapse-free survival and **(B)** breast cancer–specific survival in all cases.

**Figure 3:**
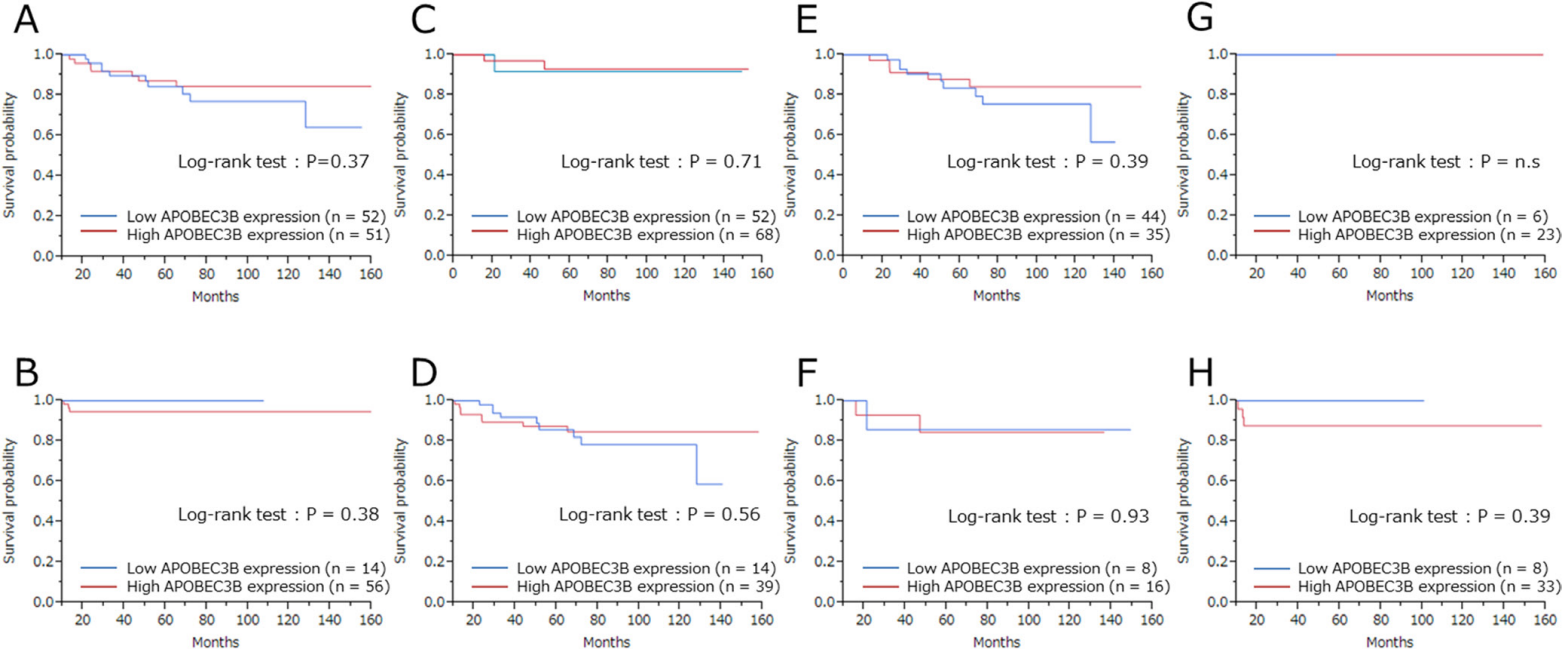
Relapse-free survival (RFS) according to APOBEC3B mRNA expression among the different subtypes Kaplan–Meier plots showing the association of APOBEC3B mRNA expression with RFS. **(A)** ER+ cases, **(B)** ER- cases, **(C)** HER2+ cases, **(D)** HER2- cases, **(E)** Luminal (ER+ and/or PgR+, HER2-) cases, **(F)** Luminal-HER2 (ER+ and/or PgR+, HER2+), **(G)** HER2-enriched (ER- and PgR-, HER2+), **(H)** triple-negative (ER-, PgR- and HER2-) cases.

**Figure 4:**
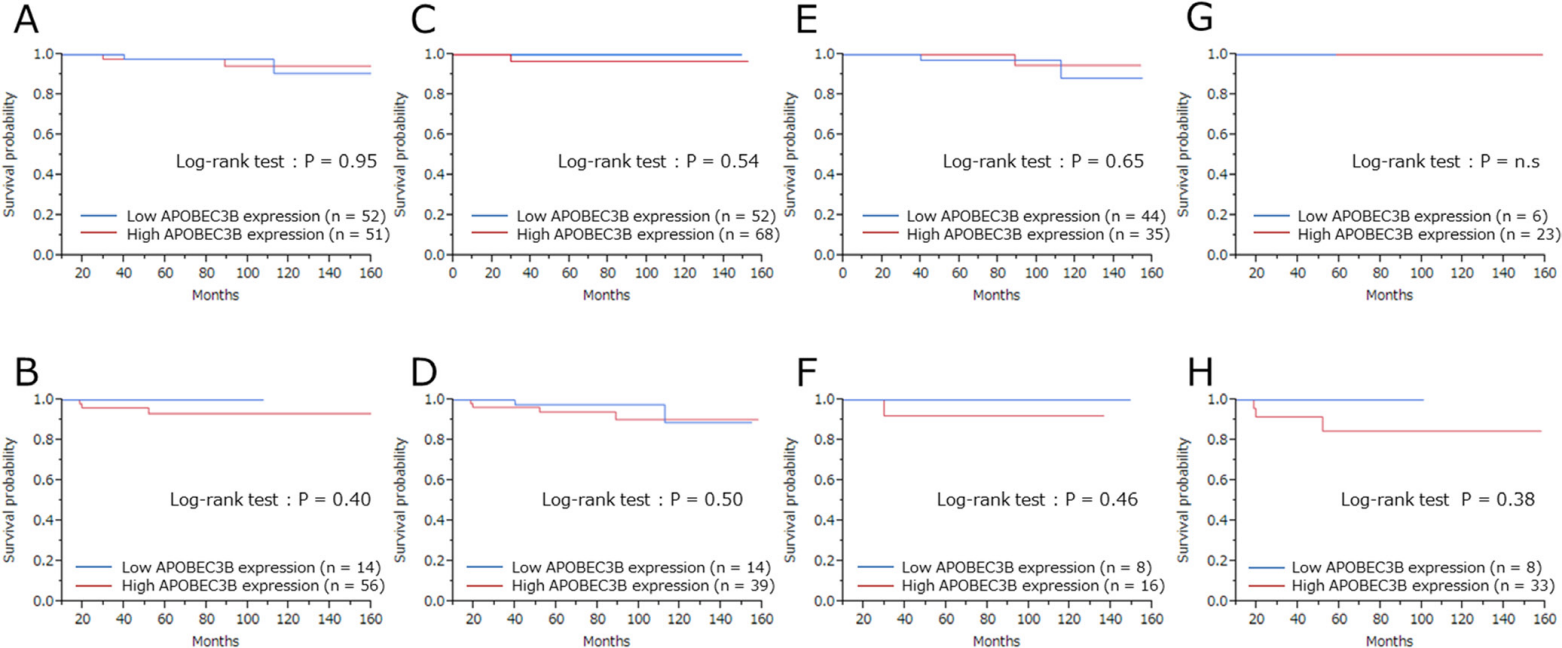
Breast cancer–specific survival (BCSS) according to APOBEC3B mRNA expression among the different subtypes Kaplan-Meier plots showing the association of APOBEC3B mRNA expression with BCSS. **(A)** ER+ cases, **(B)** ER- cases, **(C)** HER2+ cases, **(D)** HER2- cases, **(E)** Luminal (ER+ and/or PgR+, HER2-) cases, **(F)** Luminal-HER2 (ER+ and/or PgR+, HER2+), **(G)** HER2-enriched (ER- and PgR-, HER2+), **(H)** triple-negative (ER-, PgR- and HER2-) cases.

Finally, we investigated prognostic relevance of APOBEC3B classification (high/low) and pCR for all patients receiving NAC. In the analysis of relapse-free survival (RFS), local recurrences and distant metastases were considered as events (median follow-up 63 months). Among 20 recurrent cases, there were 12 cases of distant metastases and 8 cases of local recurrence. Seven patients died as a result of breast cancer, and these were regarded as events when analyzing breast cancer–specific survival (BCSS). There was no correlation between patient outcome and APOBEC3B mRNA expression (Supplementary Figure 3). We found no statistically significant association between RFS (P = 0.24) or BCSS (P = 0.61) and APOBEC3B expression (Figure 2). Similarly, there was no statistically significant correlation between RFS or BCSS and APOBEC3B expression among the different breast cancer subtypes (Figures 3, 4).

## DISCUSSION

In this study, we found that APOBEC3B mRNA expression levels correlated with the efficacy of chemotherapy. Moreover, high APOBEC3B mRNA expression was a predictive factor for pCR and APOBEC3B mRNA expression level did not correlate with breast cancer prognosis for patients receiving NAC.

Predicting pCR is very important for patient prognosis and for a therapy plan. To date, several multigene assays like Oncotype Dx [35], PAM50 [36, 37], MammaPrint [38] and 95 GC [39] have been developed and reconstructed for predicting prognosis and deciding adjuvant chemotherapy to improve prognosis, but predictors of pCR have not yet been established. In our study, high APOBEC3B mRNA expression levels positively correlated with pCR. This is the first report showing the correlation between APOBEC3B mRNA expression and therapeutic sensitivity to NAC. Furthermore, multivariate analysis showed APOBEC3B as an independent predictive factor for pCR as well as ER status and HER2 status. In stratified analysis, APOBEC3B was also a significant predictive factor for pCR in HER2- and triple negative subgroups (Table 3). According to our findings, ER and HER2 status were reported as predictive factors for pCR [40–44]. Ki-67 labeling index has also been reported as a predictor of pCR in several studies [28, 45–47]. Although a significant association was found in univariate analysis in our study, its significance was lost in multivariate analysis (Table 3), probably due to a positive correlation between Ki-67 labelling index and APOBEC3B (Spearman coefficient = 0.39; P = <.0001). Since APOBEC3B is related to tumor proliferation (Ki-67 labelling index) [21], tumors with high APOBEC3B expression might be sensitive to chemotherapy. The mechanism correlating tumor proliferation and APOBEC3B with chemotherapy efficacy has not been elucidated in our study and future research is required.

Recently, a difference has been shown in pCR rate after NAC in breast cancer by the intrinsic subtypes; patients with HER2-enriched or triple-negative tumors are more likely to achieve pCR than those with a luminal-type tumor [32, 33]. In our study, we observed a substantial difference in pCR rate among tumor subtypes, which was almost consistent with the results of other published studies [32, 33]. Therefore, predicting pCR by APOBEC3B expression in luminal type offers a useful opportunity for NAC selection. However, in our study APOBEC3B mRNA expression was not associated with pCR in luminal type. Obtaining pCR for luminal type is difficult because of the hormone susceptibility and the low proliferative potential [48]. In our study, ER expression inversely correlated with Ki67 (Spearman coefficient = -0.45; P = <.0001) and APOBEC3B expression (Spearman coefficient = -0.41; P = <.0001). The association between pCR and APOBEC3B might have been low for luminal type.

Then we evaluated the relationship between APOBEC3B mRNA expression and clinicopathological characteristics. High APOBEC3B mRNA expression was significantly related to high nuclear grade, high Ki67 labeling index, negative ER status and positive HER2 status, as reported in recent studies (Table 1). These parameters are progressive factors, and this result is consistent with previous studies showing high APOBEC3B mRNA expression in many progressive cases. Previous studies have shown that high APOBEC3B mRNA expression was associated with poor prognosis [21-25, 49, 50]. In particular, it was reported that high APOBEC3BmRNA expression was an independent prognostic factor for ER+ and lymph node-negative cases [21, 23, 25]. However, our study could not reveal the association between APOBEC3B mRNA expression levels and prognosis, in part because of the difference in patient population among trials. Also, we think that the prognosis of patients with high APOBEC3B mRNA expression might be improved by NAC because those patients had higher sensitivity to chemotherapy. Although there are reports showing similar results in terms of prognosis between preoperative chemotherapy and postoperative chemotherapy, the discrepancy could be attributed to the fact that all patients in our study received NAC but not all the patients in the other studies received NAC [26, 27].

Our study has some limitations. First, this is a retrospective study. When we selected the patients, who were candidate to receive adjuvant chemotherapy at diagnosis, patients with high responsiveness to chemotherapy might have been selected and the result might have been affected by the selection bias. We need to confirm and validate this result using data from other facilities. Second, discrepancies of prognosis between our study and previous ones were due to the timing of adjuvant chemotherapy. Because previous studies had examined the prognosis of postoperative patients with adjuvant therapy but not with NAC, there is no study comparing prognosis of patients with neoadjuvant chemotherapy and that of patients with adjuvant chemotherapy in terms of APOBEC3B mRNA expression.

In conclusion, here we demonstrate the relationship between APOBEC3B mRNA expression and sensitivity to NAC, and its role as a predictive factor for pCR in breast cancer patients receiving NAC. This is a novel finding about APOBEC3B and its potential use as a surrogate marker for pCR. Although APOBEC3B mRNA expression was not associated with breast cancer prognosis, the prognosis of patients with high APOBEC3B mRNA expression might be improved by NAC. We believe that our findings are relevant for planning an effective therapy.

## PATIENTS AND METHODS

### Patients and tumor material

Breast tumor specimens from 274 female patients with primary invasive breast carcinoma (stage I, II and III) who received treatment and surgery at Kumamoto University Hospital between 2004 and 2016 were included in this study. The median age of the patients was 53 years (range 20–78) and median duration of follow-up was 63 months. Informed consent was obtained from all patients. The ethics committee of Kumamoto University Graduate School of Medical Sciences approved this study protocol. All patients had undergone pretreatment biopsies using core needle biopsy or vacuum assisted biopsy with a 14G needle and were diagnosed with invasive breast carcinoma before NAC. Biomarkers were analyzed using pretreatment specimens, and the patients were treated with at least 4 courses (commonly up to 8 courses) of NAC such as anthracycline and/or taxane-containing regimens. Neoadjuvant treatment was administered depending on clinical practice guidelines of the Japanese Breast Cancer Society on the primary therapy of early breast cancer according to tumor biology (ER, PR, HER2, Ki67 labeling index) [51, 52]. We based our evaluation on the Reporting Recommendations for Tumor Marker Prognostic Studies (REMARK) criteria [53]. The representative regimens of chemotherapy were as follows: FEC (5-fluorouracil 500 mg/m^2^, epirubicin 100 mg/m^2^, and cyclophosphamide 500 mg/m^2^, every 3 weeks) followed by docetaxel (75 mg/m^2^, every 3 weeks) or paclitaxel (80 mg/m^2^, every week) each for 4 cycles; EC (epirubicin 90 mg/m^2^, and cyclophosphamide 600 mg/m^2^, every 3 weeks) followed by docetaxel (75 mg/m^2^, every 3 weeks) or paclitaxel (80 mg/m^2^, every week) each for 4 cycles; TC (docetaxel 75 mg/m^2^ and cyclophosphamide 600 mg/m^2^, every 3 weeks) for 6 cycles and FEC for 6 cycles. Trastuzumab was added in combination with chemotherapy in 71.7% of all HER2-positive patients.

### Evaluation of treatment response

The response of primary breast cancer during NAC was evaluated using clinical diagnostic imaging (ultrasound and magnetic resonance imaging). The achievement of pCR on postoperative specimens was defined as no evidence of residual invasive tumor in the breast or axillary lymph nodes. Noninvasive breast residuals were allowed (pCR: ypT0/ypTis).

### Total RNA extraction, real-time quantitative reverse-transcription polymerase chain reaction (RT-qPCR)

All tissue samples had previously been fixed in 10% neutral-buffered formalin for up to a maximum of 24 h. Total RNA was extracted from 4 FFPE sections (5 μm); the tumor compartment was selectively hollowed out with a sterilized blade, using the AllPrep DNA/RNA/miRNA Universal Kit (QIAGEN, Venlo, the Netherlands) in accordance with the manufacturer’s instructions. Total RNA quantification was measured by a NanoDrop 2000 spectrophotometer (Nano-Drop Technologies, Wilmington, DE, USA), determined based on the A260/A280 absorbance ratio. Total RNA (0.5 μg) was reverse-transcribed to complementary DNA (cDNA) by using PrimeScript^®^ RT Master Mix (Takara Bio, Otsu, Japan), in accordance with the manufacturer’s procedure. RT-qPCR was performed using the comparative method based on the Taq-Man chemistry on the ABI 7900HT Fast System (Applied Biosystems, Foster City, CA, USA). RT-qPCR was carried out in a solution containing 5.0 μL of 2X TaqMan^®^ Fast Advanced Master Mix (Applied Biosystems), 0.5 μL of TaqMan Gene Expression Assay (APOBEC3B: Hs00358981_m1, b-Actin: Hs01060665_g1, PUM1: Hs00982775_m1, TAF-10: Hs00359540_g1, FKBP15: Hs00910471_m1; all the primers and probes were purchased from Applied Biosystems), 3.5μL of nuclease-free water and 1.0 μL of cDNA sample (10 ng/μL) in a total volume of 10 μL. The maximum cycle threshold (Ct) value was set at 40. Negative controls were included in each run. Relative mRNA levels were determined from the threshold cycle for amplification using the ΔΔCt method by SDS 2.2 software (Applied Biosystems). Determination of Ct values was performed in duplicate and normalized to the Ct values of simultaneous duplicate measurements of the expression of 4 housekeeping genes (b-Actin, PUM1, TAF-10 and FKBP15 from the same samples) by Data Assist_ software (Applied Biosystems). These housekeeping genes were selected based on our previous study [54].

### Immunohistochemical analysis

All tissue samples had previously been fixed in 10% neutral-buffered formalin for up to a maximum of 72 h. Histological sections (4 μm) were deparaffinized and incubated for 10 min in methanol containing 0.3% hydrogen peroxide to block endogenous peroxidase. They were then immunostained with rabbit monoclonal antibodies against ERα (SP1, Ventana Japan, Tokyo, Japan), PgR (1E2, Ventana Japan) and HER2 (4B5, Ventana Japan). To detect the expression of these antibodies, we used the NexES IHC Immunostainer (Ventana Medical Systems, Tucson, AZ, USA) in accordance with the manufacturer’s instructions. ER and PgR were evaluated by percentage of nuclear staining (0–100%), and samples were considered positive when more than 1% of the nucleus was stained. HER2 expression was determined by IHC staining and evaluated using the same method as the HercepTest (Dako Japan, Tokyo, Japan); membranous staining was scored on a scale of 0 to 3+. According to the 2013 ASCO/CAP guidelines, we considered a tumor to be HER2+ when the specimen either scored ≥3+ by IHC or showed a HER2/CEP17 ratio with more than 2.0-fold increase in HER2 gene amplification (determined by dual color *in situ* hybridization using Ventana Inform HER2 Dual ISH HER2 kits [Roche Diagnostics Japan, Tokyo, Japan]) according to the manufacturer’s instructions [55, 56]. Ki67 was scored according to the percentage of nuclear stained cells out of all cancer cells in the hot spot of the tumor, regardless of the intensity, in a ×400 high-power field (Ki67 labeling index [57]). We counted between 500 and 1,000 tumor cells as recommended by the International Ki67 in Breast Cancer Working Group [58].

### Statistical analysis

The nonparametric Wilcoxon test was adopted for statistical analysis of association between APOBEC3B mRNA expression and pCR status. The best cut-off point of pCR for APOBEC3B mRNA expression levels was determined through a receiver operating characteristics (ROC) curve and used for classification of APOBEC3B mRNA expression. The association between APOBEC3B mRNA expression status and clinicopathological factors was evaluated using Chi-square or Fisher’s exact test. Logistic regression methods were also adopted for univariate and multivariate analyses to assess the associations of clinical and biological parameters with pCR. Odds ratios (ORs) and 95% confidence intervals (CIs) were calculated. Relapse-free survival and breast cancer–specific survival curves were calculated according to the Kaplan–Meier method and verified by the log-rank test. A statistically significant difference was defined at P < 0.05. All statistical analyses were performed using JMP software version 11 for Windows (SAS Institute Japan, Tokyo, Japan).

## SUPPLEMENTARY MATERIALS FIGURES AND TABLE


