# Supplementary material for: APOBEC3B gene expression as a novel predictive factor for pathological complete response to neoadjuvant chemotherapy in breast cancer
Source: Oncotarget. 2018 Jul 17;9(55):30513–26. doi: 10.18632/oncotarget.25495 (PMC6078135; doi:10.18632/oncotarget.25495)
Supplement: Supplementary file 1 [file oncotarget-09-30513-s001.pdf]

APOBEC3B gene expression as a novel predictive factor for pathological complete response to neoadjuvant chemotherapy in breast cancer

SUPPLEMENTARY MATERIALS

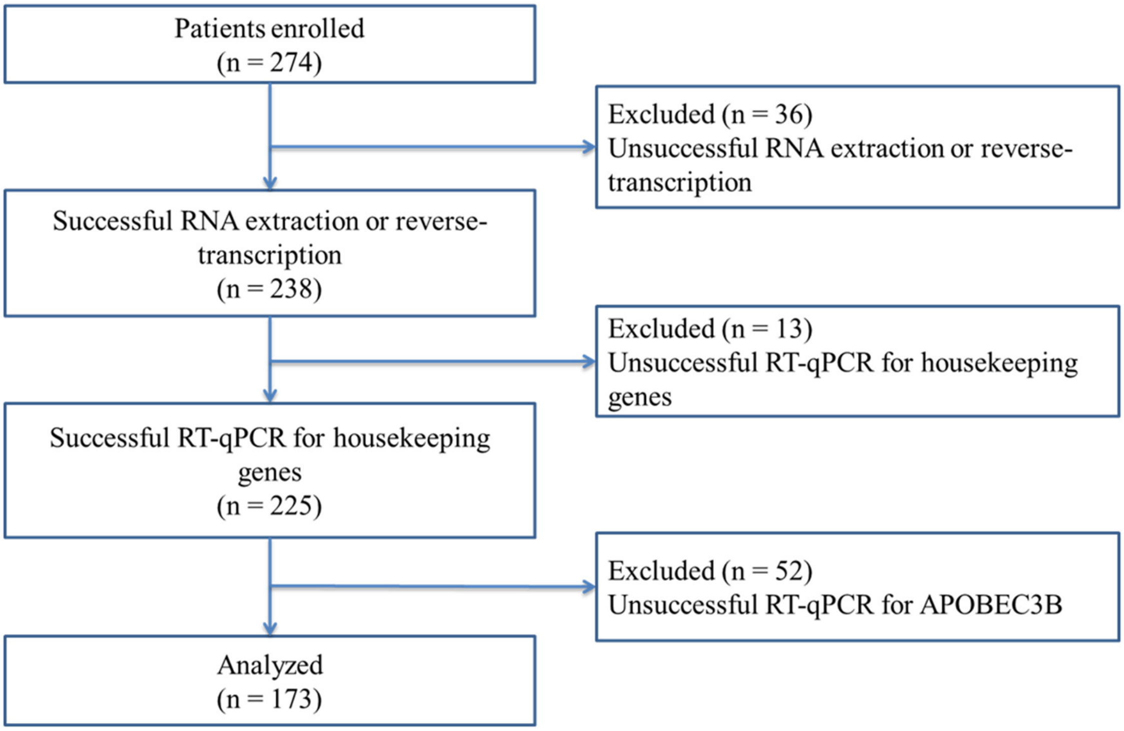

Supplementary Figure 1: Diagram of patient enrollment and population analysis. Two hundred and seventy-four primary breast cancer patients receiving NAC in more than 4 courses and undergoing surgery at our institute were enrolled in this retrospective study.

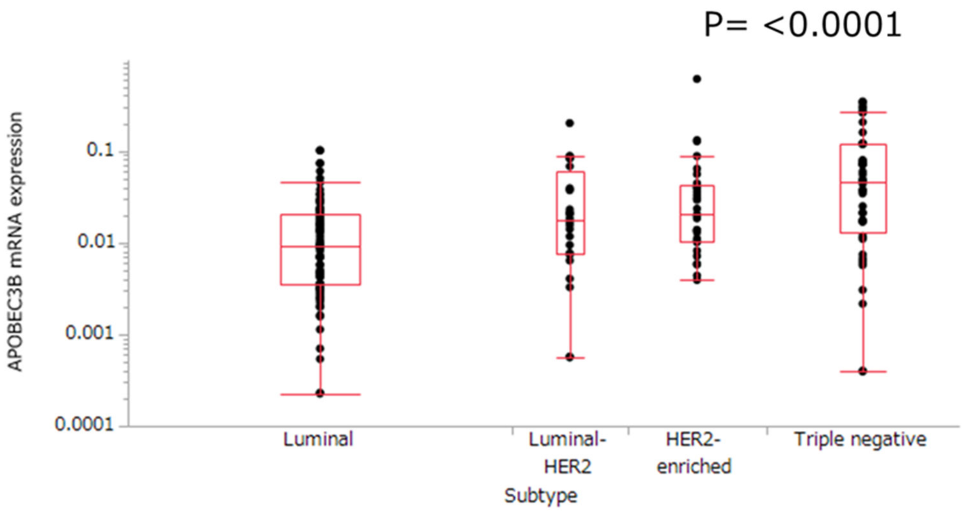

Supplementary Figure 2: Correlation between APOBEC3B mRNA expression and subtypes. Relationship of APOBEC3B mRNA expression withsubtypes verified by the Wilcoxon test. Luminal (ER+ and/or PgR+, HER2-), Luminal-HER2 (ER+ and/or PgR+, HER2+), HER2-enriched (ER- and PgR-, HER2+), Triple negative (ER-, PgR- and HER2-).

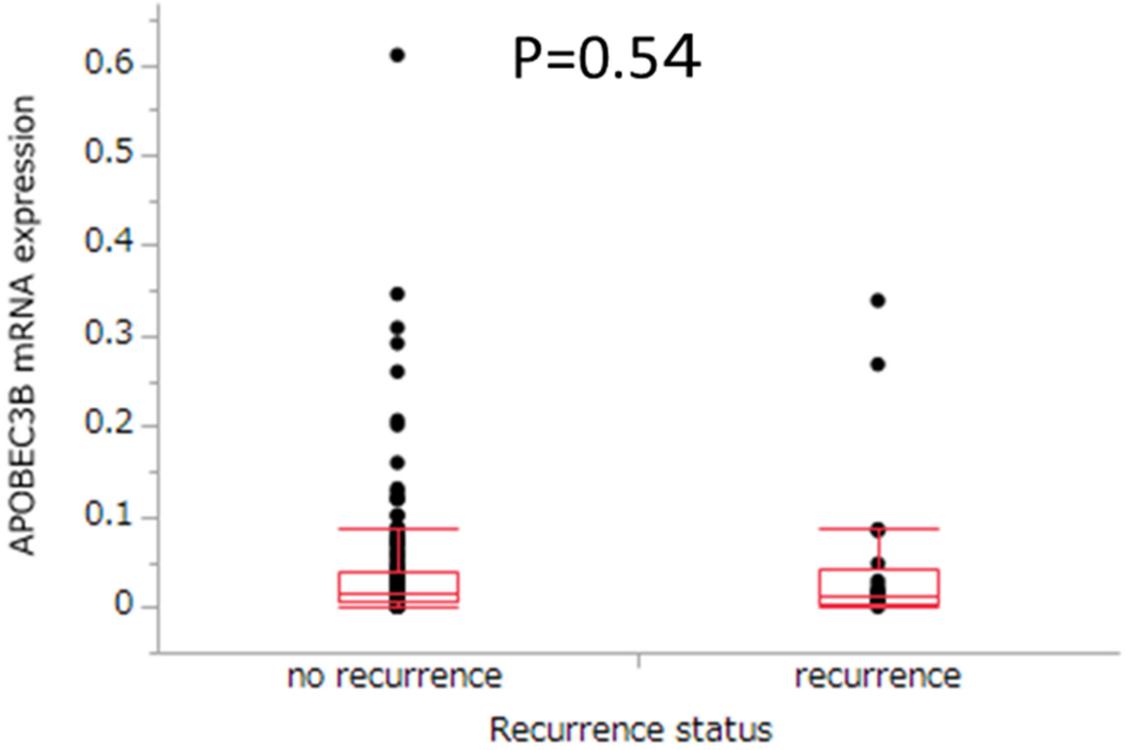

Supplementary Figure 3: Correlation between APOBEC3B mRNA expression and recurrence. Relationship of APOBEC3B mRNA expression with recurrence verified by the Wilcoxon test.

Supplementary Table 1: Patient clinicopathological characteristics (n = 173)

Characteristics	Number of patients (%)
Age at biopsy	
<50	71 (41.0%)
≥50	102 (59.0%)
Median (range)	53 (24-78)
Menopausal status	
Premenopausal	72 (41.6%)
Postmenopausal	101 (58.4%)
Tumor size (mm)	
<20	29 (16.8%)
≥20	144 (83.2%)
Nuclear Grade	
1	34 (19.7%)
2	63 (36.4%)
3	76 (43.9%)
Ki67 labeling index	
<20	26 (15.0%)
≥20	147 (85.0%)
Median (range)	
Nodal status	
Negative	51 (29.5%)
Positive	122 (70.5%)
Stage	
I	12 (7.8%)
II	108 (64.1%)
III	53 (28.1%)
Tumor subtype	
ER+/HER2-	79 (45.6%)
ER+/HER2+	24 (13.9%)
ER-/HER2+	29 (16.8%)
ER-/HER2-	41 (23.7%)
Neoadjuvant chemotherapy treatment	
Anthracycline and taxane	110 (63.6%)
Docetaxel and cyclophosphamide	10 (5.8%)
Trastuzumab and chemotherapy	38 (22.0%)
Other	15 (8.6%)
Adjuvant chemotherapy treatment	
Only endocrine therapy	79 (45.6%)
Endocrine therapy and trastuzumab	24 (13.9%)
Only trastuzumab	29 (16.8%)
None	41 (23.7%)
pCR status	
pCR	63 (36.4%)
non-pCR	110 (63.6%)

ER; estrogen receptor, PgR; progesteron receptor, HER2; human epidermal growth factor 2, SD; standard deviation, pCR; pathological complete response.
